# The PPAR****α****/****γ**** Agonist, Tesaglitazar, Improves Insulin Mediated Switching of Tissue Glucose and Free Fatty Acid Utilization *In Vivo* in the Obese Zucker Rat

**DOI:** 10.1155/2013/305347

**Published:** 2013-10-27

**Authors:** Kristina Wallenius, Ann Kjellstedt, Pia Thalén, Lars Löfgren, Nicholas D. Oakes

**Affiliations:** AstraZeneca R&D Mölndal, 431 83 Mölndal, Sweden

## Abstract

Metabolic flexibility was assessed in male Zucker rats: lean controls, obese controls, and obese rats treated with the dual peroxisome proliferator activated receptor (PPAR) *α*/*γ* agonist, tesaglitazar, 3 **μ**mol/kg/day for 3 weeks. Whole body glucose disposal rate (*R*_*d*_) and hepatic glucose output (HGO) were assessed under basal fasting and hyperinsulinemic isoglycemic clamp conditions using [3,^3^H]glucose. Indices of tissue specific glucose utilization (*R*_*g*_′) were measured at basal, physiological, and supraphysiological levels of insulinemia using 2-deoxy-D-[2,6-^3^H]glucose. Finally, whole body and tissue specific FFA and glucose utilization and metabolic fate were evaluated under basal and hyperinsulinemic conditions using a combination of [U-^13^C]glucose, 2-deoxy-D-[U-^14^C]glucose, [U-^14^C]palmitate, and [9,10-^3^H]-(R)-bromopalmitate. Tesaglitazar improved whole body insulin action by greater suppression of HGO and stimulation of *R*_*d*_ compared to obese controls. This involved increased insulin stimulation of *R*_*g*_′ in fat and skeletal muscle as well as increased glycogen synthesis. Tesaglitazar dramatically improved insulin mediated suppression of plasma FFA level, whole body turnover (*R*_*fa*_), and muscle, liver, and fat utilization. At basal insulin levels, tesaglitazar failed to lower HGO or *R*_*fa*_ compared to obese controls. In conclusion, the results demonstrate that tesaglitazar has a remarkable ability to improve insulin mediated control of glucose and FFA fluxes in obese Zucker rats.

## 1. Introduction

Impaired trafficking of fatty acids between oxidative disposal in nonadipose tissues and storage in adipose tissue, leading to ectopic lipid accumulation, may be causative in impaired glucose control [1], dyslipidemia [2], and inflammation [3], all important factors in the etiology of type 2 diabetes and cardiovascular disease. Metabolic flexibility is defined as the ability to switch from predominantly lipid metabolism, with high fluxes of fatty acids in the fasting state, to enhanced glucose uptake, oxidation and storage under hyperinsulinemic conditions [4]. Impaired metabolic flexibility could in theory induce ectopic lipid accumulation either via defective control of fatty acid availability due to insulin resistance [5, 6] or impaired fatty acid oxidation capacity [7, 8] or both. 

Whatever the precise pathophysiological mechanism, it has been shown that lowering fatty acid availability by suppressing 24 h FFA levels, using short term intensive treatment with the nicotinic acid analog acipimox, induced substantial improvements in blood glucose control in patients with type 2 diabetes [9]. While this validates the pharmacodynamic principle of FFA lowering, unfortunately tachyphylaxis towards the FFA lowering effect prevents this nicotinic acid analog from providing a robust and durable therapy for improving glucose control [9, 10]. An alternative approach to reducing ectopic lipid accumulation might be to enhance local fatty acid oxidation. This could be done either by forcing FFA oxidation (e.g., by inhibiting acetyl-CoA carboxylase 2 [11]) or by increasing the capacity to oxidize fatty acids (e.g., by increasing mitochondrial mass) potentially allowing larger physiological increments in oxidation. The approach of increasing fatty acid oxidation to achieve improved glucose control is however speculative because of the potential for accelerated lipid oxidation to inhibit glucose metabolism [12], and final judgment about the usefulness of such a pharmacodynamic principal awaits critical clinical evidence. 

Peroxisome proliferator-activated receptor (PPAR) *γ* agonists, thiazolidinediones, are used for the treatment of type 2 diabetes [13], and PPAR*α* agonists belonging to the fibrate class of compounds are used for the treatment of dyslipidemias [14]. Here we wanted to test the concept that a combined PPAR*α*/*γ* agonist could improve metabolic flexibility in a widely used animal model of insulin resistance and dyslipidemia associated with obesity, the fa/fa Zucker rat. For this purpose we used tesaglitazar which binds and activates PPAR*α* and PPAR*γ* [15] and was under clinical investigation for its ability to correct disorders of glucose and lipid metabolism. We previously established that this agent increases whole body glucose metabolic insulin action in the obese Zucker rat [16, 17] but the tissue locus of this effect has not been reported. Using tracer methods, the present study provides a comprehensive insight into *in vivo* glucose and FFA fluxes, as well as, metabolic fate at the whole body and individual tissues levels. The results demonstrate both a profound metabolic inflexibility in the untreated obese Zucker rats compared with the lean control animals and a remarkable ability of tesaglitazar to restore insulin mediated fuel switching in these animals.

## 2. Materials and Methods

### 2.1. Animals

Experimental procedures were approved by the Local Ethics Review Committee on Animal Experiments (Göteborg region). Male, 8-wk-old Zucker rats (Charles River Wiga GmbH, Suffield, Germany) were individually housed in a temperature- (20–22°C) and humidity-controlled (40–60% RH) facility with a 12 h light-dark cycle (lights on 06:00) and had free access to rodent chow (R3, Lactamin AB, Stockholm, Sweden) and tap water. At the time of the acute tracer studies (see below), rats were 12–15 wks of age. 

### 2.2. Groups

The following groups of lean (Fa/?) and obese (fa/fa) Zucker rats were studied: lean untreated controls (Lean), obese untreated controls (Obese), and obese treated with tesaglitazar 3 *μ*mol/kg/day (Tesaglitazar) for 3 weeks. This dose gives a close to maximal plasma glucose, insulin and triglyceride lowering in ob/ob mice [16]. Tesaglitazar was dosed daily at 13:00 for 3 wk by oral gavage. Untreated control rats were also gavaged according to the same schedule with an equal volume of vehicle (0.5% carboxymethyl cellulose, 2.5 mL/kg).

### 2.3. Preparation of Animals for Tracer Experiments

On the day of the tracer experiments animals were placed in a clean cage, without food but with continued access to water, at 07:00 h, and the final substance/vehicle gavage was given. Animals were weighed and anesthetized at ~09:00 h (thiobutabarbital sodium salt, Inactin, RBI, Natick, MA; lean 120, obese 180 mg*·*kg^−1^, i.p.). Rats were tracheotomized with PE 240 tubing and breathed spontaneously. One catheter (PE 50 tubing) was placed in a carotid artery for blood sampling and recording of arterial blood pressure and heart rate. Up to five catheters (PE 10) were placed together in one jugular vein for infusions of tracers, insulin, and glucose and for administering top-up doses of anesthetic, if needed. The catheters were filled with Na citrate solution (20.6 mM) in normal saline to prevent clotting. The arterial catheter patency was maintained by continuous infusion of Na citrate (20.6 mM in saline, 5 *μ*L/min) from shortly after carotid catheterization until the conclusion of the experiment. Body temperature was monitored using a rectal thermocouple and maintained at 37.5°C by means of servo controlled external heating. A stabilization period of ~150 min elapsed between completion of the surgical preparation and commencement of metabolic studies at ~12:00 h.

### 2.4. Isoglycemic-Hyperinsulinemic Clamp Studies

The target clamp blood glucose level was determined for each animal to be equal to its own basal blood glucose level (average of at least 3 stable consecutive samples over a 20 min period commencing at least 130 min after completion of surgery). Human insulin (Actrapid, Novo Nordisk, Bagsvaerd, Denmark) was infused at a constant rate based on lean body mass estimated according to [17] via a dedicated jugular vein catheter using a syringe pump (CMA 1100, Carnegie Medicin, Solna, Sweden). Arterial blood glucose was measured every 5 min using a glucose analyzer (YSI Incorporated, Yellow Springs, OH, USA; 15 *μ*L blood per sample). Blood glucose was clamped at the basal level (isoglycemia) with a variable rate infusion of 20% (w : v) glucose, using a syringe pump (Model 22 I/W, Harvard Apparatus Inc., South Natic, MA, USA) via a dedicated jugular vein catheter. Steady state in both blood glucose level (within ±10% of the target level) and glucose infusion rate (GIR) was generally achieved within 60 min of clamp start. 

Clamps were performed at different plateau levels of hyperinsulinemia from physiologically relevant to supraphysiological. Those denoted ClampL were performed at levels of hyperinsulinemia that were physiologically relevant for the particular group under study. In this case insulin infusion rates were tailored for each of the three groups of Zucker rats to produce plateau insulin levels corresponding to those observed in the freely fed postprandial state (2 h after onset of the dark phase) as determined in separate experiments: Lean 30, Obese 120, and Tesaglitazar 60 pmol/kg_lbm_/min. To obtain information about insulin responsiveness ClampH clamps were performed at supraphysiological levels of hyperinsulinemia, with insulin infused at 10 times the corresponding ClampL rates for each group, that is, Lean 300, Obese 1200, and Tesaglitazar 600 pmol/kg_lbm_/min. An additional ClampM was performed only in the Tesaglitazar group with an insulin infusion rate at 210 pmol/kg_lbm_/min to achieve a plasma insulin level corresponding to the postprandial levels seen in the Obese group.

### 2.5. Study Outlines

*Study 1: Effects of Hyperinsulinemia in the Physiological Range on Whole Body Glucose Disposal *(*R*_*d*_)* and Hepatic Glucose Output (HGO)*. Each animal was studied during a basal phase, immediately followed by a clamp phase.

*Basal Phase*. D-[3,^3^H]glucose (^3^H-glucose, Amersham Pharmacia Biotech, Uppsala, Sweden) was administered as an initial 2 min priming infusion (~30 × 10^6^ dpm/kg) followed by a constant infusion (1 × 10^6^ dpm/min/kg). Arterial blood samples (~100 *μ*L) were collected at 40, 60, 70, and 80 min following commencement of tracer administration directly from the carotid catheter into potassium EDTA containing tubes (Microvette CB300, Sarstedt, Nümbrecht, Germany), for determination of plasma ^3^H-glucose (see below) and plasma glucose levels using a YSI 2700 glucose analyzer (YSI Incorporated, Yellow Springs, OH, USA). Samples collected at 60, 70, and 80 min were used to define the basal phase plasma ^3^H-glucose steady state and the subsequent target blood glucose level for the clamp state. 

*Clamp Phase*. Beginning after collection of the 80 min sample, insulin was infused at rates that were needed to perform ClampL clamps (defined previously). Arterial blood glucose was clamped at the basal level using the methods described previously. Arterial blood samples collected at ~120, 140, 150, and 160 min following commencement of the tracer administration were used to estimate the clamp phase ^3^H-glucose steady state. Additional plasma (~50 *μ*L) was collected (during the basal phase at 80 min and during the clamp phase at 160 min) and stored at −20°C for later determination of glucose, insulin, free fatty acids (FFA), and triglycerides (TG).

*Study 2: Insulin Action in the Nonhepatic Tissues*. The dependence of tissue specific glucose utilization (*R*_*g*_′) on insulin was assessed in Lean, Obese, and Tesaglitazar groups by making separate studies in the basal state (Basal), as well as under ClampL and ClampH conditions (defined previously). In addition the Tesaglitazar group was studied under ClampM conditions. 

Isoglycemic-hyperinsulinemic clamp studies were performed according to the methods detailed previously. 2-deoxy-D-[2,6-^3^H]glucose (^3^H-2DG, Amersham Pharmacia Biotech) was administered as an intravenous bolus (~8 × 10^7^ dpm) following attainment of clamp steady state, 60 min after commencement of the insulin infusion (clamp studies), or in the case of basal studies after collection of the basal blood sample. Blood samples for determination of plasma tracer concentration were collected according to the sampling schedule described in [18]. Estimates of the whole body rate of glucose disposal (*R*_*d*_′) were obtained from the plasma disappearance of ^3^H-2DG, after correction for urinary excretion [19]. *R*_*g*_′ was calculated from the tissue accumulation of ^3^H-label, essentially as described in [18]. Immediately following collection of the final blood sample (45 min after tracer administration) rats were given an overdose of thiobutabarbital (120 mg/kg). Samples of the following tissues were collected: white quadriceps muscle (WQ), red quadriceps (RQ), red gastrocnemius (RG), epididymal adipose tissue (white fat), interscapular brown adipose tissue (brown fat), diaphragm, heart, and cerebellum. For measurement of total ^3^H-label content, freshly collected tissue pieces were weighed and placed in small cardboard cones for combustion (see below). Two blood samples (150 *μ*L each) were collected; plasma was separated and stored at −20°C for later determination of steady state plasma glucose, insulin, and FFA concentrations: one was collected just prior to the tracer bolus, the other 45 min after the tracer bolus. 

*Study 3: Metabolic Flexibility.* In order to assess the influence of physiological changes in insulin level on metabolic switching of glucose and FFA fuels, animals were studied in either the Basal or ClampL states (defined previously). In this study a number of whole body and tissue specific glucose and FFA fluxes were evaluated: an estimate of *R*_*d*_, (${\hat{R}}_{d}$), rate of plasma FFA appearance (*R*_*fa*_), *R*_*g*_′, glycogen synthesis rate (*R*_gly_), tissue FFA utilization index (*R*_f_*), and rate of FFA incorporation into storage (*R*_*fs*_). The tracer experiment began following collection of a basal blood sample (Basal study) or attainment of clamp steady state (Clamp study). A bolus (~400 *μ*L) containing D-(U-^13^C) glucose (^13^C-glucose, ~6 mg, Cambridge Isotope Laboratories, Inc., Andover, MA) and 2-deoxy-D-[U-^14^C] glucose (^14^C-2DG, ~5 × 10^7^ dpm, Amersham, Solna, Sweden) was injected intravenously via a dedicated jugular catheter and arterial blood samples (75 *μ*L) were collected at 2, 5, 10, 15, 20, and 25 min. Then at 30 min an albumin-palmitate-fatty acid tracer complex was infused via another dedicated jugular catheter at 230 *μ*L/min for a total of 4 min as described in [20]. Two fatty acid tracers were included; (1) the partially-metabolizable analogue [9,10-^3^H]-(R)-2-bromopalmitate (^3^H-R-BrP, ~5 × 10^7^ dpm, synthesized and purified as described in [21]) and (2) [U-^14^C]palmitic acid (^14^C-P, ~2.5 × 10^7^ dpm; Amersham, Solna, Sweden). Additional arterial blood samples (75 *μ*L) were collected at 31, 32, 33, 34, 35, 36, 38, 42, and 46 min with respect to the glucose tracer bolus. Blood samples were centrifuged at 4°C, and two plasma aliquots were pipetted: 25 *μ*L into 2 mL extraction mixture (for determination of ^14^C-2DG, ^13^C-glucose, ^3^H-R-BrP, and ^14^C-P levels) and 10 *μ*L onto a cellulose cone for combustion (for determination of total ^3^H and ^14^C-levels, see below). After collection of the final blood sample rats were given an overdose of thiobutarbital, and tissues were rapidly dissected and frozen in liquid N_2_. Tissue samples were stored at −80°C awaiting analysis for tracer content (see below) and in the case of red skeletal muscle, glycogen content as well.

### 2.6. Determination of Tissue and Plasma Tracer Concentrations 

*Study 1*. Plasma ^3^H-glucose concentration was determined by pipetting 25 *μ*L freshly collected plasma directly into ice chilled 100 *μ*L 0.15 M ZnSO_4_. Then 100 *μ*L of 0.15 M Ba(OH)_2_ was added, the tube vortexed, and centrifuged, and an aliquot of the supernatant was counted following evaporation (70°C, N_2_), reconstitution in 0.5 mL water and addition of 5 mL scintillant.

*Study 2*. Plasma ^3^H-2DG concentration and tissue ^3^H-activity were determined based on the production of ^3^H_2_O by completely oxidizing the 25 *μ*L plasma samples and ~100 mg tissue pieces using a Packard System 387 Automated Sample Preparation Unit (Packard Instrument Co. Inc., Meriden, CT). 

*Study 3*. Freshly collected plasma (25 *μ*L) was pipetted directly into glass test tubes with 2 mL isopropanol : heptane : 1 M acetic acid (40 : 10 : 1 vol). Plasma extraction was performed according to [22] with the exception that sulphuric acid was replaced with 1 M acetic acid. ^14^C-P and ^3^H-R-BrP, as well as esterified fatty acids, were quantitatively removed in the upper, heptane phase, while ^13^C-glucose and ^14^C-2DG, as well as polar metabolites and ^3^H_2_O, were quantitatively recovered in the lower aqueous phase. Upper phase polar lipids (including ^14^C-P and ^3^H-R-BrP) were separated from neutral lipids (including endogenously esterified ^14^C-P) by means of solid phase extraction (200 mg NH_2_ columns, Isolute, Sorbent AB, Göteborg, Sweden). Retained polar lipids were driven off the columns using 5% glacial acetic acid in methyl-tert-butyl ether. The effluent was mixed with scintillant and counted for determination of ^14^C-P and ^3^H-R-BrP. Two aliquots of the lower phase were taken: one counted for total ^14^C-content (used to determine ^14^C-2DG); a second for determination of ^13^C-glucose. For the latter aliquot, glucose was derivatized to aldonitrile penta acetate using hydroxylamine in pyridine and acetic anhydride. The glucose derivative was dissolved in ethyl acetate after evaporation to dryness and analysed by gas-chromatography-isotope ratio-mass spectrometry using a Hewlett Packard 6890 gas chromatogram (GC) connected to an isotope ratio mass spectrometer (Finnigan Delta Plus, Finnigan, San Jose, CA) via a Finnigan GC Combustion III interface. The ^13^C/^12^C ratio of the formed CO_2_ was compared with results for a ^13^C-glucose enriched linear calibration curve and tracer to tracee ratios (TTR) for ^13^C to ^12^CO_2_ were calculated. Tissue samples were homogenized and extracted in ice chilled glass-glass homogenizers, essentially according to [23]. The organic phase was quantitatively recovered for determination of ^3^H- and ^14^C-labelled lipids. Aliquots of the aqueous phase were taken for determination of (1) total polar ^3^H- and ^14^C-labelled metabolites and (2) ^14^C-labelled anion, by solid phase extraction (200 mg PE-AX columns, Isolute) assumed to be phosphorylated ^14^C-2DG. Separate tissue pieces were used for determination of glycogen content based on the glycogen precipitation (KOH/ethanol) method and enzymatic conversion to glucose [24]. Aliquots of the resulting glucose solution were taken for (1) measurement of glucose concentration using the colorimetric kit method and (2) analysis of ^13^C-glucose as described previously for plasma.

Except for samples generated by the oxidizer, the scintillant used to make the cocktails for counting was Optiphase Hisafe III (Perkin Elmer, Upplands Väsby, Sweden). Sample disintegrations per minute were estimated using a Wallac 1409 counter (Wallac OY, Turku, Finland).

### 2.7. Measurement of Plasma Lipids, Glucose, and Insulin

Colorimetric kit methods were used to measure plasma FFA (NEFA C, Wako, Richmond, VA), and glucose (Glucose HK, Roche, Stockholm, Sweden). Plasma TG was also determined using a colorimetric method by measuring the glycerol released by complete enzymatic hydrolysis of triglycerides (Triglycerides/GB, Boehringer Mannheim, Indianapolis, IN). Colorimetric measurements were performed on a centrifugal analyzer (Cobas Bio, F. Hoffmann-La Roche & Co., Basle, Switzerland). Radioimmunoassays were used to measure plasma concentrations of insulin (Rat Insulin RIA Kit, Linco Research Inc., St. Charles, MO, USA). 

### 2.8. Calculations 

*Study 1.* *R*_*d*_ was estimated, assuming attainment of steady state, from the measured plateau plasma ^3^H-glucose concentration (*c*_*P*_) and corresponding measured plasma glucose concentration (*C*_*P*_) using *R*_*d*_ = *I* × *C*_*P*_/*c*_*P*_ where *I* is tracer infusion rate. The reported basal *R*_*d*_ value represents the average of the 60, 70, and 80 min estimates. The reported clamp *R*_*d*_ value was calculated from the average of the 140, 150, and 160 min estimates. During the basal phase, HGO was calculated as HGO = *R*_*d*_. During the clamp phase HGO was calculated as HGO = *R*_*d*_ − GIR, where GIR is the steady state glucose infusion rate. 

*Study 2.* An index of the whole body rate of glucose disposal (*R*_*d*_′) was calculated from the plasma disappearance of ^3^H-2DG according to [19]. *R*_*g*_′ was calculated from plasma kinetics of ^3^H-2DG and tissue retention of phosphorylated ^3^H-2DG based on the methods described in [18]. Note that *R*_*g*_′ data for the liver is not presented due to poor metabolic trapping of 2DG in this tissue [25]. Tissue level insulin action was assessed quantitatively in a particular group by fitting *R*_*g*_′ versus plasma insulin level ([Ins]) results for individual animals to a dose-response curve; that is,

(1) $$\begin{array}{c} R_{g}^{\prime }=R_{g}^{\prime }\left(\min\right)+\frac{\left(R_{g}^{\prime }\left(\max\right)-R_{g}^{\prime }\left(\min\right)\right)}{\left(1+10^{(\log{\text{EC}}_{50}-\log[\text{Ins}])b}\right)}, \end{array}$$

where *R*_*g*_′(min⁡) is the asymptotic value of *R*_*g*_′ estimated as [Ins]→0, *R*_*g*_′(max⁡) is the asymptotic value of *R*_*g*_′ as [Ins]→*∞*, EC_50_ is the [Ins] required to affect a half maximal response; that is, (*R*_*g*_′(min⁡) + *R*_*g*_′(max⁡))/2, *b* is a slope factor). Note that EC_50_ is the strict quantitative definition of insulin sensitivity, and the difference, *R*_*g*_′(max⁡)–*R*_*g*_′(min⁡), is the insulin responsiveness.

*Study 3.* Plasma FFA clearance rate (*K*_*fa*_) and appearance rate (*R*_*fa*_) were calculated from plasma ^14^C-P kinetics according to previously described methods [20]. Indices of utilization rates of plasma substrate by tissues (*R*), specifically *R*_*g*_′, *R*_gly_, *R*_f_*, and *R*_*fs*_ were calculated based on ^14^C-2DG, ^13^C-glucose, ^3^H-R-BrP, and ^14^C-P, respectively from the general relation:

(2) $$\begin{array}{c} R=\frac{C\times m^{*}}{\int_{0}^{T}c^{*}\left(t\right)dt}, \end{array}$$

where *C* is the plasma substrate concentration (glucose or FFA), *t* is time, *T* is the time of tissue collection, m* is the tissue accumulation of tracer product at *t* = *T*, and c*(*t*) is the plasma tracer concentration. 

${\hat{R}}_{d}$ was obtained from the plasma disappearance of ^13^C-glucose/^12^C-glucose ratio above natural abundance $\left(\hat{c}\right)$ based on the following equation:

(3) $$\begin{array}{c} {\hat{R}}_{d}=\frac{\text{Dose}}{\int_{0}^{\infty }\hat{c}dt}, \end{array}$$

where Dose is the bolus dose of ^13^C-glucose (in *μ*mol), $\hat{c}$ was calculated from the equation $\hat{c}=16÷6\times (\text{TTR}-{\text{TTR}}_{\text{na}})$, TTR_na_ = 0.0112372, and the integral was calculated using best fit parameters obtained by fitting a double exponential to the disappearance data. This estimate of ${\hat{R}}_{d}$ does not have the same accuracy as the gold standard tracer steady state method applied in Study 1.

The whole body and tissue specific glucose and FFA flux indices for all studies are summarized in Table 1.

### 2.9. Statistics

Analysis of group data was based on contrasts to specifically test the principle a priori questions of the study: Lean versus Obese (obesity effect) and Tesaglitazar versus Obese (treatment effect). Analysis of Study 3 was based on detection of the above between group difference in the basal state and the responses to insulin (ClampL-Basal). The latter was interpreted as a measure of metabolic flexibility. Statistical significance of the contrasts were evaluated on the basis of *F*-tests using the program SPSS (SPSS Inc., Chicago, IL). Results are reported as means ± SE. *P* < 0.05 was considered statistically significant.

## 3. Results

### 3.1. Study 1: Effects of Hyperinsulinemia, in the Physiological Range, on Whole Body Glucose Disposal and HGO

Three groups of age matched animals were studied: vehicle treated lean (Lean) and obese Zucker (Obese) controls and obese Zuckers treated with tesaglitazar, 3 *μ*mol/kg/day for 3 weeks (Tesaglitazar). During the treatment period body weight gain was greater in Obese compared to Lean, 4.5 ± 0.3 versus 2.3 ± 0.1 g/day, respectively; *P* < 0.001 and was further increased in Tesaglitazar to 8.3 ± 0.8 g/day (*P* < 0.01 versus Obese). Following the 3-week treatment period whole body glucose turnover was studied under basal fasting and ClampL conditions of physiological hyperinsulinemia (see Section 2). Body weights at the time of study as well as steady state glucose levels and GIRs are summarized in Table 2. This data confirms a large tesaglitazar induced increase in whole body insulin action consistent with earlier work [16]. 

**Figure 1:**
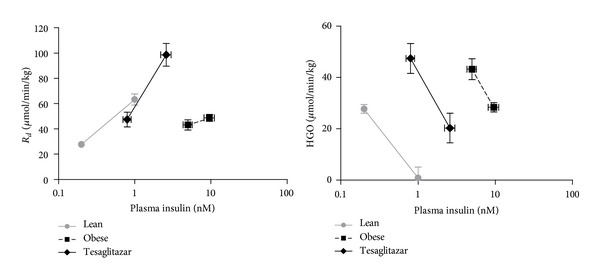
Peripheral glucose disposal (*R*_*d*_) and hepatic glucose output (HGO) in Lean, Obese and Tesaglitazar groups of Zucker rats. Data points represent mean ± SE (*n* = 4-5).

Figure 1 shows the results of the tracer based measurements, whole body glucose disposal (*R*_*d*_), and hepatic glucose output (HGO) plotted against plasma insulin at basal (fasting) and ClampL levels. Tesaglitazar treatment did not correct the elevation in basal HGO or the associated moderate hyperglycemia of the obese Zucker rat. The afore mentioned treatment induced improvement in whole body insulin action involved both enhanced peripheral insulin action (greater increment in *R*_*d*_ at lower insulin levels compared to Obese) and hepatic insulin sensitization (Figure 1). Estimated insulin concentrations required to suppress HGO to 50% of the basal level (EC_50_) were substantially reduced in Tesaglitazar 2.4 ± 0.4 versus Obese 15.4 ± 5.0 nM, *P* < 0.05. For comparison the EC_50_ for Lean was 0.65 ± 0.08 nM. 

### 3.2. Study 2: Insulin Action in the Nonhepatic Tissues

We next wanted to identify the nonhepatic tissues responsible for the tesaglitazar induced enhancement in peripheral insulin action, as well as the roles of insulin sensitivity versus responsiveness. For this purpose, *in vivo* tissue specific glucose utilization rates (*R*_*g*_′) were assessed using the 2-deoxyglucose tracer method. Dependence of *R*_*g*_′ on plasma insulin level was determined by performing studies in the basal fasting state and during glucose clamps performed at physiologic and superphysiological levels of hyperinsulinemia in Lean, Obese, and Tesaglitazar groups.  Table 3 summarizes insulin infusion rates and steady state plasma insulin and glucose levels, as well as the GIRs needed to maintain isoglycemia, with results consistent with previous findings and in addition showing that tesaglitazar treatment completely restored insulin's ability to maximally stimulate GIR (responsiveness). 

**Figure 2:**
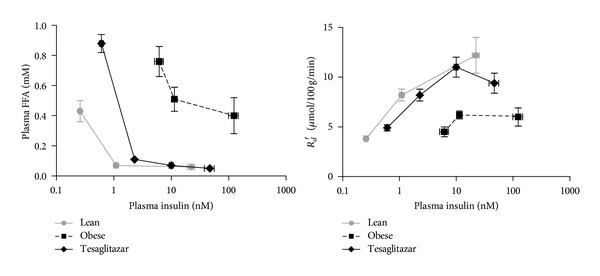
Whole body effects of insulin, under basal and hyperinsulinemic clamp conditions: suppression of plasma FFA and stimulation of glucose disposal rate *R*_*d*_′ ( = “average” tissue utilization rate) in Lean, Obese, and Tesaglitazar groups of Zucker rats. Data points represent means ± SE (*n* = 5–7).

Figure 2 shows insulin's effects on plasma FFA level and whole body *R*_*d*_′. Tesaglitazar treatment did not correct the elevation in basal FFA but induced a remarkable amelioration in the defects in insulin mediated suppression of plasma FFA and stimulation of *R*_*d*_′ involving both increased responsiveness (maximum effect) and sensitivity (leftward shift) to insulin.

**Figure 3:**
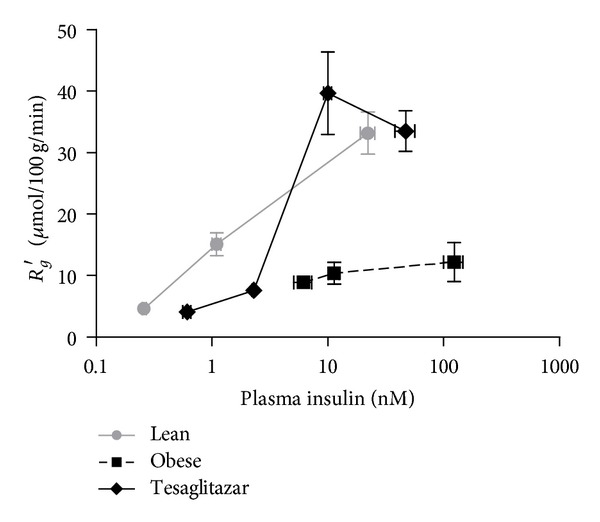
*In vivo* glucose utilization rate (*R*_*g*_′) in red quadriceps muscle as a function of plasma insulin levels under basal and hyperinsulinemic clamp conditions in Lean, Obese, and Tesaglitazar groups. Data points represent means ± SE (*n* = 5–7).

Indices of glucose utilization rates (*R*_*g*_′) in individual tissues are summarized in Table 4. In the basal state, *R*_*g*_′ of the red hindlimb muscle of the Tesaglitazar group was lower than in the Obese group, representing a normalization towards the values in the Lean group. White adipose tissue *R*_*g*_′, elevated in Obese versus Lean, was further elevated by tesaglitazar. In all insulin responsive tissues examined (i.e., not cerebellum) tesaglitazar markedly improved the ability of insulin to concentration dependently stimulate *R*_*g*_′ in the obese Zucker rat. An example showing the dependence of *R*_*g*_′ on plasma insulin is illustrated in Figure 3 for the RQ muscle. This treatment effect was so profound that it enabled quantitative estimation of insulin sensitivity (EC_50_ value for *R*_*g*_′ as defined in Section 2) in a number of tissues of the obese rat which was generally not possible in untreated animals due to a virtual absence of insulin effect. For the Tesaglitazar treated group the EC_50_ for white fat, diaphragm and RG were 0.7 nM (95% CI 0.4–1.0), 1.7 nM (1.1–2.7), and 3.5 nM (0.6–21.4), respectively. (For comparison the EC_50_ values in Lean animals were for white fat, diaphragm, and RG 0.5 nM (95% CI 0.4–0.7), 0.9 nM (0.7–1.2), and 1.4 nM (0.9–2.1), resp.). By contrast in Obese only in white fat could an estimate of EC_50_ be made: 9.6 nM (6.4–14.6). The improvement in insulin action with tesaglitazar treatment was also manifested in a general restoration of insulin responsiveness with *R*_*g*_′ at supraphysiologic insulin levels substantially higher in skeletal muscle and white fat (Figure 3 and Table 4) in Tesaglitazar compared to Obese. In the Tesaglitazar group, maximal insulin effects on both whole body glucose metabolism and *R*_*g*_′ in insulin responsive tissues were actually generally achieved under ClampM conditions, performed at insulin levels equivalent to those seen postprandially in untreated obese Zuckers (Tables 3 and 4).

### 3.3. Study 3: Insulin Mediated Switching of Glucose and FFA Fluxes

In the final study whole body and tissue specific FFA metabolism in the three groups were examined in both the basal fasting state and under ClampL conditions of hyperinsulinemia (see Section 2). Furthermore, to evaluate substrate switching in the same experiment, glucose fluxes were also assessed along with FFA fluxes. For this purpose, four tracers were applied in each animal to provide virtually simultaneous information about plasma FFA utilization (*R*_f_*), plasma FFA incorporation into storage (*R*_*fs*_), and glucose utilization (*R*_*g*_′) in a number of tissues. In addition an estimate of glucose incorporation into glycogen synthesis (*R*_gly_) was obtained for red skeletal muscle.  Table 5 summarizes the whole body parameters for Study 3. The insulin and glucose metabolic data (including an estimate of *R*_*d*_) are consistent with results from Studies 1 and 2, confirming the insulin resistant phenotype of the obese Zucker as well as its amelioration with tesaglitazar.

*Whole Body FFA Metabolism.* *R*_*fa*_ was substantially elevated in Obese compared to Lean confirming an earlier study [26] showing mobilization, rather impaired clearance, to be the main mechanism for fatty acid elevation in obese animals (Table 5). Tesaglitazar's influence on systemic FFA availability was highly dependent on prevailing insulin level. In the basal state plasma FFA levels were similar in Tesaglitazar and vehicle treated obese rats. Interestingly in this situation tesaglitazar treated animals actually exhibited elevated *R*_*fa*_ compared to vehicle controls which did not translate into a further elevation in plasma FFA levels due to a treatment induced increase in *K*_*fa*_ (Table 5), the combined ability of the tissues of the body to take up FFA. Tesaglitazar profoundly improved insulin's ability to suppress systemic FFA mobilization (*R*_*fa*_), the main mechanism of treatment induced FFA lowering. ClampL FFA levels in the Tesaglitazar group were in fact virtually normalized despite a 2.3-fold higher *R*_*fa*_ compared to the Lean group, due to the treatment induced enhancement in *K*_*fa*_ (Table 5). 

**Figure 4:**
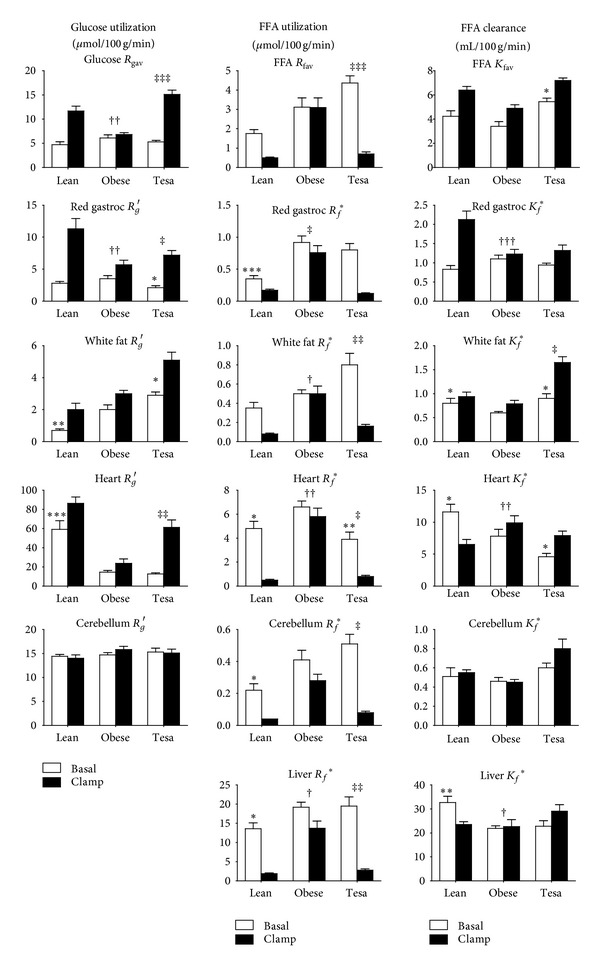
Whole body turnover and tissue specific utilization rates of glucose and FFA. Glucose and FFA flux data are shown in the left and right hand panels, respectively, under basal (open histograms) and hyperinsulinemic clamp (closed histograms) conditions. Mean ± SEM (*n* = 6-7). *P < 0.01, **P < 0.01, and ***P < 0.001 versus Obese basal state. ^†^ *P* < 0.05, ^††^ *P* < 0.01 insulin effect in Obese versus Lean (based on Clamp-Basal differences). ^‡^ *P* < 0.05, ^‡‡^ *P* < 0.01, and ^‡‡‡^ *P* < 0.001 insulin effect in Tesaglitazar (Tesa) versus Obese (based on Clamp-Basal differences).

*Tissue Specific Metabolism.* Figure 4 summarizes tissue specific rates of glucose utilization *R*_*g*_′ (left panel), FFA utilization *R*_f_* (middle), and FFA clearance *K*_f_* (right). In the top row of this figure are “average tissue” glucose and FFA utilization rates (R_gav_ and *R*_fav_) which were obtained from the ${\hat{R}}_{d}$ and *R*_*fa*_ data presented in Table 5 corrected for body weight. This data confirms that tesaglitazar treatment markedly ameliorated the impairment in insulin mediated switching of glucose and fatty acid utilization in the obese Zucker at the whole body level. Most importantly, a similar pattern is reflected in the *R*_*g*_′ and *R*_f_* profiles for muscle and white fat showing the quantitative importance of these tissues to whole body metabolism. 

*Tissue Specific FFA Utilization, R*_f_*. A couple of general features in the data shown in the middle column, Figure 4, are worthy of comment. Firstly, independent of group, the magnitude of *R*_f_* varies widely between the tissues with liver values 2 orders of magnitude higher than those for the cerebellum. Secondly, a similar between group pattern of *R*_f_* was consistently seen in all of the tissues which mirror plasma FFA levels (see Table 5) reflecting the strong influence of substrate supply in driving FFA utilization. Basal *R*_f_* values tended to be higher in Obese animals versus Lean, with little effect of tesaglitazar except in the heart (which showed a lowering, normalization of *R*_f_*). In Obese, there was a widespread failure of insulin to suppress *R*_f_* in strong contrast to the situation in Lean and Tesaglitazar. 

*Tissue Specific FFA Clearance*, *K*_f_*. *K*_f_* is a measure of the local ability of the tissues to utilize FFA. While FFA level was clearly a dominant factor in determining between group differences in *R*_f_* of an individual tissue, the *K*_f_* data (right most column, Figure 4) revealed insulin mediated regulation/dysregulation of FFA utilization at the local tissue level. Thus in Lean tissues, insulin exerted heterogeneous effects on *K*_f_* which were lost in the Obese tissues: stimulation in skeletal muscle and suppression in heart and liver with no significant effect in white fat or cerebellum. Surprisingly, tesaglitazar did not induce normalization of the response to insulin in this parameter in any of the tissues examined. In white fat tesaglitazar induced an increase in *K*_f_* which was potentiated by insulin (Figure 4). 

*Tissue Specific Nonoxidative FFA Disposal, R*_*fs*_. While *R*_f_* indexes uptake into both oxidative and nonoxidative disposal, the parameter *R*_*fs*_ (summarized in Table 6) represents flux into nonoxidative disposal only. Generally the between group pattern described previously for *R*_f_* was also seen for *R*_*fs*_ with these two variables strongly positively correlated (data not shown). In the basal state, tesaglitazar normalized *R*_*fs*_ in the heart only with no apparent normalization of the elevated levels in other tissues including skeletal muscle and liver (which in fact was further increased by treatment). This latter result was particularly surprising to us due to the established influence of PPAR*α* stimulation in upregulating *β*-oxidation machinery. Tesaglitazar greatly improved insulin's ability to suppress tissue nonoxidative FFA disposal resulting in normalization of *R*_*fs*_ at physiological hyperinsulinemia in all tissues examined. 

*Glucose Utilization by Individual Tissues, R*_*g*_′. Overall the *R*_*g*_′ results (left column, Figure 4) generally agree with Basal and ClampL results from Study 2 consistent with tesaglitazar induced amelioration of insulin resistance in key insulin target tissues. Moderate differences in absolute *R*_*g*_′ levels between the two studies may have been the result of the different methodologies used to assess tissue 2DG tracer retention (see Section 2) with a potentially more accurate method used in the current study. Note that hepatic *R*_*g*_′ data is not represented in Figure 4 as the estimates are not valid due to poor metabolic trapping of 2DG in this tissue [25]. 

*Glycogen Synthesis in Red Muscle, R*_gly_. Net glycogen mass and synthesis (*R*_gly_), obtained by averaging red gastrocnemius and red quadriceps muscle data, are summarized in Table 6. *R*_gly_ was close to zero in all groups in the basal fasting state. Tesaglitazar succeeded in completely normalizing the insulin induced increase in *R*_gly_. Comparison of the magnitudes of *R*_*g*_′ (reflecting the sum of glycolysis and glycogen synthesis) and *R*_gly_ suggests that the insulin induced increases in *R*_*g*_′ in Lean and Tesaglitazar groups involved increases in both glycolysis and glycogen synthesis. In the basal state, tesaglitazar reduced glycogen mass to the levels seen in lean animals (Table 7).

## 4. Discussion

The whole body glucose and FFA metabolic responses to hyperinsulinemia in lean and obese Zucker rats shown in this study are in many respects analogous to the differences reported a number of years ago between patients with type 2 diabetes and control subjects [27] confirming the relevance of the obese Zucker rat as a preclinical model. Results of the present study extend the metabolic characterization of the obese Zucker rat revealing impaired switching of fatty acid and glucose utilization at the tissue level. In lean Zucker rats, physiological hyperinsulinemia substantially reduced the flux of FFA from plasma into skeletal muscles of diverse fiber composition, heart, adipose tissue, and liver. In strong contrast, raising insulin levels in obese Zucker rats failed to quench fluxes of FFA into the tissues. 

It has been shown that tesaglitazar ameliorates insulin resistance in animal models [16, 17], as well as centrally obese/hypertriglyceridemic nondiabetic humans [28, 29] and patients with type 2 diabetes [30]; however, the tissue loci of these effects have not been reported. The present study demonstrates that the tesaglitazar induced whole body insulin sensitization was due to a widespread restoration of insulin action. Thus tesaglitazar greatly increased insulin's ability to suppress HGO (Figure 1) and also restored insulin's ability to stimulate skeletal muscle glucose uptake (Table 4). This latter action was of a profound nature with untreated obese Zuckers lacking any robust dose response relationship between skeletal muscle glucose uptake and insulin level. Treatment succeeded in restoring a clear dose response relationship including a remarkable increase in the maximal insulin responsiveness in skeletal muscle (Figure 3). Not only did tesaglitazar enhance insulin stimulated skeletal muscle glucose uptake but also normalized the rate of insulin stimulated glycogen synthesis in this tissue to that seen in the lean Zuckers (Table 7), albeit at a higher insulin level. The effects of tesaglitazar on augmenting insulin mediated control of hepatic and skeletal muscle glucose metabolism are consistent with the results of a number of studies with other PPAR*γ* agonists on glucose metabolism in both animal models [31] and even patients with type 2 diabetes [32]. 

Tesaglitazar also augmented insulin's ability to quench systemic FFA availability and decrease FFA uptake, as well as incorporation into nonoxidative disposal in skeletal muscles, heart and the liver. Putting these effects together with the afore mentioned effects on glucose metabolism, it is apparent that tesaglitazar induced an effective and widespread improvement in the ability of insulin to switch fuel mixture in the tissues. This is illustrated in Figure 4 by the results at the level of the whole body, skeletal muscle, heart, and adipose tissue. Under basal insulin conditions, obese Zucker rats exhibited elevated rates of FFA utilization and storage in the majority of tissues driven by elevated circulating FFA levels, disturbances that were not affected by tesaglitazar (discussed below). The apparent failure of the substance to enhance tissue level FFA oxidation in the basal state (as suggested by unaltered FFA uptake in combination with unaltered FFA incorporation into nonoxidative disposal in skeletal muscle and liver) surprised us based on our expectation that tesaglitazar should increase fatty acid oxidation capacity via PPAR*α* activation suggested by previous studies showing tesaglitazar induced hepatic enlargement and upregulation of CYP4A [16, 17]. To further explore the specific potential of PPAR*α* activation to enhance *in vivo* FFA oxidation we treated rats with a selective PPAR*α* agonist, WY14,643. Despite a substantial (>2-fold increase) in ex vivo FFA oxidation capacity in the liver we detected no WY14,643 induced enhancement of *in vivo* FFA oxidation in the basal fasting situation or under conditions of substantially elevated oxidative metabolism induced by the uncoupler dinitrophenol (unpublished observations). On the basis of these results and our recent finding that plasma FFA oxidation is not reduced in obese compared to lean Zucker rats [33], it appears that levels of FFA oxidation, FFA oxidation capacity, and insulin resistance are independent, at least in this model. This is in contrast to the situation reported by some studies in insulin resistant humans where reduced fasting levels of whole body fatty acid oxidation are associated with impairment in skeletal muscle fatty acid oxidation capacity [4].

Many of the beneficial effects of tesaglitazar on glucose and lipid metabolism may be secondary to its effect on FFA exchange in adipose tissue. Specifically, fatty acid overflow into nonadipose tissues is ameliorated by two mechanisms: an enhanced ability of fat tissue to take up FFA [34], as well as a greater ability of postprandial insulin levels to suppress FFA appearance rate (Figure 4, middle panel). This latter effect is probably a result of more effective suppression of FFA release from adipocytes, but perhaps also due to reduced spillover of products of intravascular lipolysis into the systemic circulation. Similar effects on adipose tissue FFA exchange have been observed with a thiazolidinedione [26] but were not observed with a selective PPAR*α* agonist (unpublished observations). Whether these actions are mediated via PPAR*γ* agonism or alternatively more recently suggested molecular targets of thiazolidinediones [35, 36] remains to be determined.

The treatment induced reduction in flux of FFA into hepatic lipid stores at high physiological insulin levels (Table 6) in these habitually hyperphagic/hyperinsulinemic animals would tend to lower local TG synthesis and hepatic TG secretion. Indeed we have previously reported that tesaglitazar (same dose and treatment period as the current study) potently lowers hepatic TG content and hepatic TG secretion in obese Zucker rats [17]. In man a reduction in VLDL TG production is expected to have important antidyslipidemic effects [37]. Reduced hepatic FFA availability might also be a mechanism for the improvement in insulin suppression of HGO [38]. The consequences of lowering fatty acid supply is however unlikely to be limited to the liver. Thus, the marked tesaglitazar induced reduction in FFA flux into skeletal muscles (at hyperinsulinemia) may explain the apparent enhancement in insulin stimulated oxidative glucose disposal in this tissue via the glucose-fatty acid cycle [12]. Furthermore, the reduced muscle FFA flux might also lower accumulation of lipid intermediates potentially reducing interference with insulin signaling [39] which may be the basis for the enhanced insulin stimulated glucose uptake and glycogen synthesis rates. 

The effects of tesaglitazar on glucose and lipid metabolism in the obese Zucker rat [16] have generally proven to be predictive of effects in patients with type 2 diabetes [30] and obese/insulin resistant subjects [28, 29, 40], with improvements in glucose tolerance, lipid tolerance, insulin sensitivity, and meal induced FFA lowering, as well as decreased plasma TG levels and decreased apoCIII levels seen in both humans and rodents. One aspect of glucose metabolism that tesaglitazar did not correct in the obese Zucker rats was the defect in basal HGO, substantially elevated in obese compared to lean Zucker rats (Figure 1). This may have been a consequence of the failure of treatment to lower plasma FFA flux into the liver in this state (Figure 4). This was probably a result of the lack of lowering of plasma FFA level in the basal state which represents a clear departure from the response in clinical studies where overnight fasting FFAs were consistently lowered by tesaglitazar [28–30]. One possible explanation may be the relatively short treatment duration in the current study. In response to PPAR*γ* agonists adipose tissue undergoes cellular remodeling [41], with large insulin resistant fat cells emptying and newly differentiated cells filling with triglycerides before a new steady state is reached. Prior to achieving this new steady state it may be that fasting insulin levels which fall in response to developing treatment induced whole body insulin sensitization are insufficient to normalize FFA release from adipose tissue. In patients with type 2 diabetes, for example, approximately 6 weeks of treatment is required for tesaglitazar to achieve the full glucose lowering effect [30].

The clinical development of tesaglitazar was halted in large part due to concern over treatment induced increases in serum creatinine levels in patients [42]. The impressive effects of this agent on glucose and lipid control in animal models and human subjects, including data suggesting important antiatherogenic effects [43, 44], provide strong support for continuing the search for future pharmacodynamic principals that enhance metabolic flexibility.

In conclusion, we have for the first time assessed *in vivo* fluxes and metabolic fate of both glucose and FFA at the whole body and individual tissue levels as well as the control of these fluxes by insulin in the obese Zucker rat. The results reveal that defective insulin regulation of metabolism extends beyond glucose control to include profound impairments in control of tissue FFA fluxes in this animal model. In addition it was determined that the improvement in whole body insulin mediated glucose control afforded by the PPAR*α*/*γ* agonist tesaglitazar was due to restoration of insulin's ability to suppress hepatic glucose production and stimulate glucose uptake in skeletal muscle. Beyond glucose control, however, tesaglitazar restored insulin's ability to suppress fatty acid availability and the flux of FFA from plasma into muscles and liver which together with the effects on glucose metabolism resulted in a remarkable improvement in insulin mediated switching of fuel utilization.

## Figures and Tables

**Table 1:** Whole body and tissue specific fluxes of glucose and FFA.

Abbreviation	Measures	Tracer	Comment
Study 1			
* R*_*d*_	Glucose disposal rate	^ 3^H-Glucose	Best *R*_*d*_ estimate, tracer steady-state
HGO	Hepatic glucose output	^ 3^H-Glucose	HGO = *R*_*d*_ − GIR
Study 2			
* R*_*d*_′	Glucose disposal rate index	^ 3^H-2DG	Approx *R*_*d*_ value, analog transient
* R*_*g*_′	Tissue glucose utilization index	^ 3^H-2DG	
Study 3			
* * ${\hat{R}}_{d}$	Glucose disposal rate	^ 13^C-Glucose	Approx *R*_*d*_ value, tracer transient
* R*_*fa*_	FFA appearance rate	^ 14^C-P	
* K*_*fa*_	FFA clearance rate	^ 14^C-P	
* R*_*g*_′	Tissue glucose utilization index	^ 14^C-2DG	
* R*_gly_	Tissue glycogen synthesis rate	^ 13^C-Glucose	
* R*_f_*	Tissue FFA utilization index	^ 3^H-R-BrP	
* K*_f_*	Tissue FFA clearance	^ 14^C-P	
* R*_*fs*_	Tissue FFA storage	^ 14^C-P	

**Table 2:** Body weights, plasma glucose levels and steady state clamp GIRs in Lean, Obese and Tesaglitazar groups of Zucker rats used in Study 1.

	Lean	Obese	Tesaglitazar
Body weight (g)	380 ± 12	630 ± 12***	651 ± 18
Basal glucose (mM)	6.1 ± 0.4	8.8 ± 0.3**	8.7 ± 0.9
Clamp glucose (mM)	5.5 ± 0.3	8.6 ± 0.4**	8.3 ± 0.9
GIR (*µ*mol/min/kg)	62.5 ± 5.9	20.0 ± 3.5***	80.0 ± 5.7^†^

Results are expressed as mean ± SE (*n* = 4-5). **P < 0.01, ***P < 0.001 versus Lean. ^†^ *P* < 0.05 versus Obese.

**Table 3:** Insulin infusion rates, steady state plasma insulin and glucose levels, and GIRs needed to maintain isoglycemia during the clamps used in Study 2.

Group	Insulin infusion	Insulin	Glucose	GIR
Body weight (g)	(pmol/kg_lbm_/min)	(nM)	(mM)	(*µ*mol/min/kg)
Lean 396 ± 13				
Basal	0	0.3 ± 0.1	6.7 ± 0.3	0
ClampL	30	1.1 ± 0.1	6.5 ± 0.4	77.3 ± 6.8
ClampH	300	22.3 ± 3.2	6.5 ± 0.2	152.7 ± 10.9
Obese 583 ± 21***				
Basal	0	6.2 ± 1.1***	9.8 ± 0.4***	0
ClampL	120	11.4 ± 1.2***	10.0 ± 0.3***	28.3 ± 5.7***
ClampH	1200	125 ± 24**	9.7 ± 1.8	41.2 ± 13.9***
Tesaglitazar 654 ± 13^†^				
Basal	0	0.6 ± 0.1^†††^	8.8 ± 0.4	0
ClampL	60	2.1 ± 0.1^†††^	8.7 ± 0.4^†^	105.4 ± 5.8^†††^
ClampM	210	10.0 ± 0.8	9.5 ± 0.6	142.6 ± 9.6^†††^
ClampH	600	47.1 ± 9.1^††^	9.8 ± 1.1	150.5 ± 10.9^†††^

ClampL performed at a physiological (postprandial) level of hyperinsulinemia, ClampH at supraphysiological insulin levels, ClampM (Tesaglitazar group only) designed to achieve plateau insulin levels corresponding to those in Obese ClampL group. Results are expressed as mean ± SE (*n* = 5–7). **P < 0.01, and ***P < 0.001 versus corresponding state of Lean. ^†^ *P* < 0.05, ^††^ *P* < 0.01, and ^†††^ *P* < 0.001 versus corresponding state of Obese.

**Table 4:** Tissue-specific glucose utilization rates (*R*_*g*_′, *µ*mol/100 g/min) in the basal and insulin stimulated states during hyperinsulinemic-isoglycemic clamp studies in Lean, Obese, and Tesaglitazar groups of Zucker rats used in Study 2.

	WQ	RG	White fat	Brown fat	Diaphragm	Heart	Cerebellum
Lean							
Basal	1.4 ± 0.1	3.7 ± 0.4	1.0 ± 0.1	20.3 ± 6.6	13.5 ± 1.8	38.5 ± 3.9	15.1 ± 1.1
ClampL	4.9 ± 0.5	13.2 ± 1.9	2.0 ± 0.2	108 ± 40	45.9 ± 4.1	114.8 ± 8.3	16.9 ± 0.9
ClampH	13.3 ± 1.6	28.8 ± 2.9	2.4 ± 0.4	115 ± 12	67.1 ± 5.5	80.7 ± 8.5	16.5 ± 0.7
Obese							
Basal	3.0 ± 0.3**	7.4 ± 1.0**	2.9 ± 0.3**	5.8 ± 0.5**	15.7 ± 1.8	18.6 ± 1.8***	17.2 ± 1.4
ClampL	3.0 ± 0.2**	8.2 ± 1.3	4.0 ± 0.4**	10.7 ± 1.3**	23.9 ± 3.4**	27.5 ± 3.0***	20.1 ± 1.4*
ClampH	3.1 ± 0.5***	11.7 ± 3.1***	3.8 ± 0.2	11.7 ± 2.2***	24.1 ± 5.3***	56.0 ± 15	20.2 ± 0.9**
Tesaglitazar							
Basal	2.4 ± 0.2	3.8 ± 0.3^†††^	4.3 ± 0.4^††^	3.6 ± 0.2	14.3 ± 2.6	18.0 ± 2.4	14.5 ± 1.2
ClampL	3.4 ± 0.3	7.2 ± 0.6	7.2 ± 0.6^†††^	13.8 ± 3.8	51.9 ± 4.0^†††^	73.9 ± 6.3^†††^	16.7 ± 0.6^†^
ClampM	9.3 ± 1.0^‡‡‡^	41.4 ± 4.6^‡‡‡^	5.8 ± 0.7^‡^	25.3 ± 3.6^‡‡^	79.8 ± 4.9^‡‡‡^	62.5 ± 1.6^‡‡‡^	18.5 ± 1.3
ClampH	6.9 ± 0.9^†^	27.6 ± 3.3^†††^	6.9 ± 0.9^††^	31.0 ± 4.3	65.6 ± 3.7^†††^	64.7 ± 3.6	17.4 ± 0.7^†^

Results are expressed as mean ± SE (*n* = 5–7). WQ, white quadriceps muscle. RG, red gastrocnemius muscle. White fat represents the epididymal fat pad. Brown fat represents interscapular brown adipose depot. *P < 0.05, **P < 0.01, and ***P < 0.001 versus corresponding state of Lean. ^†^ *P* < 0.05, ^††^ *P* < 0.01, and ^†††^ *P* < 0.001 versus corresponding state of Obese. ^‡^ *P* < 0.05, ^‡‡^ *P* < 0.01, and ^‡‡‡^ *P* < 0.001 versus ClampL Obese group.

**Table 5:** Body weights, plasma factors, and whole body glucose and FFA metabolic turnover parameters in Lean, Obese, and Tesaglitazar groups of Zucker rats studied under basal and clamp conditions used in Study 3.

Group	Lean		Obese		Tesaglitazar	
State	Basal	ClampL	Basal	ClampL	Basal	ClampL
Body weight (g)	407 ± 11***	405 ± 20	603 ± 14	582 ± 18	666 ± 30	664 ± 27
Insulin (nM)	0.74 ± 0.16***	1.10 ± 0.10	5.88 ± 0.67	13.61 ± 2.60^†^	0.67 ± 0.07***	2.25 ± 0.10^‡^
FFA (mM)	0.42 ± 0.03***	0.08 ± 0.01	0.89 ± 0.05	0.63 ± 0.09	0.81 ± 0.04	0.10 ± 0.01^‡‡^
*K*_*fa*_ (mL/min)	17.4 ± 2.1	25.8 ± 1.5	20.7 ± 2.3	28.3 ± 1.6	38.4 ± 2.2***	47.1 ± 2.5
*R*_*fa*_ (*µ*mol/min)	7.2 ± 1.0***	2.0 ± 0.1	18.9 ± 2.8	18.1 ± 3.2	31.0 ± 3.0*	4.6 ± 0.5^‡‡‡^
Glucose (mM)	7.4 ± 0.2**	7.7 ± 0.3	11.1 ± 1.0	11.6 ± 1.3	11.8 ± 0.5	10.0 ± 0.4
GIR (*µ*mol/min)	0	26.1 ± 2.4	0	8.9 ± 2.7^†††^	0	66.9 ± 9.2^‡‡‡^
${\hat{R}}_{d}$ (*µ*mol/min)	19.0 ± 2.2**	47.6 ± 1.2	36.0 ± 3.5	41.2 ± 2.0^††^	36.2 ± 3.1	101.8 ± 6.9^‡‡^

Results are mean ± SE (*n* = 6–9). Zucker rats were studied in the 5 h fasted state. Groups of animals were studied either in the basal state or during isoglycemic clamps performed at physiological levels of hyperinsulinemia suited to each group (insulin infusion rates as for ClampL in Study 2, defined in Table 3). Basal state differences; *P < 0.05, **P < 0.01, and ***P < 0.001 versus obese. Response to insulin (ClampL-Basal) differences; ^†^ *P* < 0.05, ^††^ *P* < 0.01, and ^†††^ *P* < 0.001 Obese versus Lean, ^‡^ *P* < 0.05, ^‡‡^ *P* < 0.01, and^‡‡‡^ *P* < 0.001 Tesaglitazar versus Obese.

**Table 6:** Tissue-specific rates of FFA incorporation into storage *R*_*fs*_ (*µ*mol/100 g/min).

Group	Lean		Obese		Tesaglitazar	
State	Basal	ClampL	Basal	ClampL	Basal	ClampL
Red gastroc	0.68 ± 0.10**	0.32 ± 0.02	1.81 ± 0.44	2.19 ± 0.25	1.68 ± 0.30	0.41 ± 0.02^‡‡^
White fat	0.84 ± 0.20	0.13 ± 0.03	1.15 ± 0.14	1.15 ± 0.22	1.90 ± 0.25**	0.32 ± 0.05^‡‡‡^
Heart	4.7 ± 0.5***	4.6 ± 0.5	12.3 ± 1.6	15.3 ± 1.4	4.8 ± 0.3***	4.9 ± 0.3
Cerebellum	0.32 ± 0.02***	0.09 ± 0.01	0.64 ± 0.09	0.58 ± 0.11	0.52 ± 0.05	0.09 ± 0.01^‡‡^
Liver	24.8 ± 1.0***	5.4 ± 0.5	37.7 ± 3.9	31.1 ± 3.4^†^	45.7 ± 4.1*	7.2 ± 0.9^‡‡‡^

Results are mean ± SE (*n* = 6–8). Groups of animals were studied either in the basal state or during isoglycemic clamps performed at physiological levels of hyperinsulinemia suited to each group (insulin infusion rates as for ClampL in Study 2, defined in Table 3). Red gastroc abbreviation for red gastrocnemius. Basal state differences; *P < 0.05, **P < 0.01, and ***P < 0.001 versus obese. Response to insulin (ClampL-Basal) differences; ^†^ *P* < 0.05 Obese versus Lean and ^‡‡^ *P* < 0.01, ^‡‡‡^ *P* < 0.001 Tesaglitazar versus Obese.

**Table 7:** Glycogen content (*µ*mol/g) and synthesis rate *R*_gly_ (*µ*mol/100 g/min) in red skeletal muscle.

Group	Lean		Obese		Tesaglitazar	
State	Basal	ClampL	Basal	ClampL	Basal	ClampL
Content	33 ± 4	40 ± 2	43 ± 7	49 ± 2	33 ± 3*	41 ± 2
*R*_gly_	0.25 ± 0.05	3.53 ± 0.91	0.38 ± 0.07	1.86 ± 0.55^†^	0.20 ± 0.02	3.99 ± 0.31^‡‡^

Results are mean ± SE (*n* = 4–8). Groups of animals were studied either in the basal state or during isoglycemic clamps performed at physiological levels of hyperinsulinemia suited to each group (insulin infusion rates as for ClampL in Study 2, defined in Table 3). Data represent values obtained by averaging red gastrocnemius and red quadriceps muscles. Basal state differences; *P < 0.05 versus Obese. Response to insulin (ClampL-Basal) differences; ^†^ *P* < 0.05 Obese versus Lean, and ^‡‡^ *P* < 0.01, Tesaglitazar versus Obese.
